# Solid Organ Transplant Litigation at One of Europe’s Largest University Hospitals

**DOI:** 10.3389/ti.2024.12439

**Published:** 2024-05-01

**Authors:** Jacques Belghiti, François Cauchy, Corinne Antoine, Gérard Cheron, Marie Matignon

**Affiliations:** ^1^ Direction des Affaires Juridiques de l’Assistance Publique Hôpitaux de Paris, Assistance Publique Hôpitaux de Paris, Paris, France; ^2^ Department of Hepato-Pancreato-Biliary and Liver Transplantation, Hôpitaux Universitaires de Genève, Genève, Switzerland; ^3^ Assistance Publique Hôpitaux de Paris, Service de Néphrologie Transplantation, Hôpital Saint Louis, Paris, France; ^4^ Agence de la Biomédecine, Saint Denis, France; ^5^ Assistance Publique Hôpitaux de Paris, Service de Néphrologie et de Transplantation Rénale, Fédération Hospitalo-Universitaire « Innovative Therapy for Immune Disorders », Centre Hospitalo-Universitaire Henri Mondor, Créteil, France; ^6^ University of Paris-Est-Créteil, Institut National de la Santé et de la Recherche Médicale (INSERM) U955, Team 21, Institut Mondor de Recherche Biomédicale, Créteil, France

**Keywords:** information, solid organ transplantation, complaints, litigation, postoperative complication

## Abstract

Due to its intrinsic complexity and the principle of collective solidarity that governs it, solid organ transplantation (SOT) seems to have been spared from the increase in litigation related to medical activity. Litigation relating to solid organ transplantation that took place in the 29 units of the Assistance Publique-Hôpitaux de Paris and was the subject of a judicial decision between 2015 and 2022 was studied. A total of 52 cases of SOT were recorded, all in adults, representing 1.1% of all cases and increasing from 0.71% to 1.5% over 7 years. The organs transplanted were 25 kidneys (48%), 19 livers (37%), 5 hearts (9%) and 3 lungs (6%). For kidney transplants, 11 complaints (44%) were related to living donor procedures and 6 to donors. The main causes of complaints were early post-operative complications in 31 cases (60%) and late complications in 13 cases (25%). The verdicts were in favour of the institution in 41 cases (79%). Solid organ transplants are increasingly the subject of litigation. Although the medical institution was not held liable in almost 80% of cases, this study makes a strong case for patients, living donors and their relatives to be better informed about SOT.

## Graphical Abstract

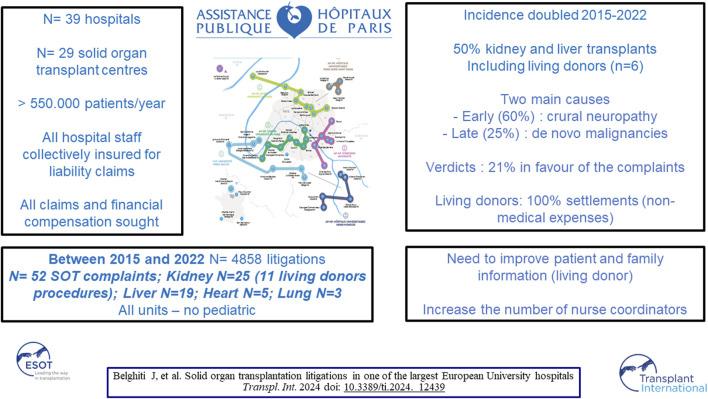

## Introduction

Solid organ transplantation (SOT) combines the best medical care with a high level of expertise involving cutting-edge medical and surgical management. This procedure saves the lives of countless patients suffering from irreversible liver, lung or heart failure, and increases the survival rate of patients suffering from kidney failure every year [1–4]. In European countries, allografts come from anonymous donors who have died without financial compensation. However, the number of candidates for organ transplants exceeds the availability of allografts and is associated with significant post-operative mortality and morbidity. As a result, the allocation rules and the failure of this procedure may be the subject of disappointment, leading patients and families to complain. In Europe, very few legal proceedings have been reported and there is a desire to maintain a positive public image of the hospital. All this has led us to consider that SOTs are not affected by the increasing judicialization of medical activity. In the absence of reliable data to support these views, we conducted a study focusing on legal proceedings following SOT at Assistance Publique Hôpitaux de Paris (AP-HP), the largest teaching hospital in France.

## Materials and Methods

### Study Sites and Procedure

This study is a quantitative, descriptive and evaluative study within the AP-HP, which is the largest university in France with more than 30 hospitals located in Paris and the suburbs, caring for more than 8 million patients a year.

In AP-HP, all employees of all hospitals, including doctors, nurses and other (paramedical) staff, are collectively insured for civil liability claims. The AP-HP is unique in that it is its own insurer and all claims are handled and defended by a single legal department called the DAJ (Département des Affaires Juridiques). The DAJ protects and defends all AP-HP employees without the need to take out additional insurance. The procedure is as follows: in cases where patients or their relatives contest hospital care after a setback, local mediation is set up. When local mediation is successful, it never leads to a settlement. If local mediation fails or if financial compensation is sought, patients or their relatives may initiate legal proceedings to obtain medical expertise. Complaints seeking compensation are judged either by a specific independent body, the CCI, the Conciliation and Compensation Chamber. This commission, chaired by a magistrate and made up of members of civil society, analyses compensation claims free of charge when the potential damage exceeds a certain severity threshold. Analysis and advice were provided by forensic experts appointed by these courts and after confrontation between the two parties: plaintiffs (patients and/or relatives) accompanied by their lawyers and defendant including hospital concerned medical doctor and their own lawyer. The verdict must determine whether the institution is guilty of misconduct or breach of duty. In most cases, verdict follows advice of forensic experts appointed by these courts. If the damage assessments exceed the severity threshold defined by law, financial compensation is payable by the hospital in the event of fault or negligence, or by the State and the National Solidarity Fund for Medical Accidents (ONIAM) in the event of therapeutic risk. In complex situations involving negligence and therapeutic risks, responsibility is shared between ONIAM and the hospital.

An average of 600 cases are recorded by DAJ every year (ranging from 503 to 702 per year over the last 10 years). As experienced in many countries, these judicial proceedings mainly involve orthopedic surgery, primary care, obstetrics -gynecology, general surgery and neurosurgery [5, 6]. APHP collects information from 29 OT centres caring out around 1,500 OT per year, including seven kidney transplant (KT) units (810 KT/year, 54%), five liver transplant units (LT) (480 LT/year, 32%), six cardiac transplant units (CT) (170 CT/year, 11%) and four pulmonary transplant units (PT) (70 PT/year, 5%). The introduction of claims management software since 2015 has enabled the authors to examine proceedings whose verdict has been recorded from 2015 to 2022.

As far as organ transplantation from living donors is concerned, the short- and long-term risks of all procedures are first explained by the medical providers and nurse coordinators. A psychological assessment is systematically carried out for all living donors. An independent committee then checks that recipients and living donors have understood the risks and are psychologically fit to harvest organs. All SOT data is closely monitored by an independent body, the Agence de la biomédecine (ABM), which provides annual reports (activity, results) on transplant activity in France and in each transplant centre.^1^

### Claims Files

All proceedings records with analysis and advice by forensic experts were reviewed and analysed by the first author, who has extensive experience in SOT (JB). Data recorded included patient age, gender, date of SOT, date of the event giving rise to complaint, mortality, incidence of other clinical events or conditions considered relevant to the litigation and court verdict. The main grounds for the plaintiffs’ complaint were categorised as follows 1) iatrogenic complication leading to SOT; 2) failure to provide timely referral; 3) graft/recipient mismatch or technical failure during the operation; 4) failure to diagnose and treat life-threatening post-operative complications in the intensive care unit (ICU); 5) acute neuropathy attributed to nerve damage during the operation; 6) lack of information about the long-term risks of the operation, including the development of malignancy. Our study meets the criteria of reference methodology MR-004, which governs the processing of personal data for the purposes of study, evaluation or research not involving the human person, as defined by the CNIL (Commission nationale de l’informatique et des libertés), which governs personal data in France. More specifically, these are studies that do not meet the definition of research involving the human person, in particular studies relating to the re-use of data. The research must be in the public interest, which is the case for our study. Our declaration number is 2232922. Our research was conducted in accordance with the Helsinki and Istanbul declarations.

### Statistical Analysis

Continuous variables are presented as medians (min-max) and were analysed using the Kruskal-Wallis or Mann-Whitney U test, as appropriate. Categorical variables were presented as numbers and percentages and were compared using the χ^2^ test or Fisher’s exact test. All statistical tests were two-tailed and a *p*-value < 0.05 was considered statistically significant in all analyses. Statistical analyses were performed using SPSS^®^ version 24.0 software (SPSS Inc., an IBM Company, Chicago, Il, United States).

## Results

### Characteristics of Plaintiffs

Of the 4,858 procedures recorded and adjudicated from January 2015 to December 2022 at the AP-HP, 52 (1.07%) concerned SOT. While the overall number of complaints remained stable, the rate of complaints regarding SOT almost doubled over the study period, from 0.71% to 1.50% (Table 1). All adult centres performing SOT within the APHP were involved. The patients were female in 22 (42%) cases and the median age was 51 (19–74) years. No paediatric case was recorded. The surgeries were performed from 2006 to 2022 and 10 (21%) more than 5 years before the procedure. The main causes of these late complaints were *de novo* malignancies (*N* = 4) and death induced by COVID-19 (*N* = 3). Among the 46 SOT candidates or recipients, death was the reason for complaint in 28 (60%) cases. KT including one combined pancreas-KT was the main SOT involved with 25 (48%) cases. Of these, 11 (44%) concerned living donor procedures, with 6 donors procedures. Other SOT complaints were as follows 19 (37.0%) for LT including one liver-kidney transplantation; five (9%) for CT and three (6%) for PT.

**TABLE 1:** Total number of SOT proceedings registered and judged among all cases and SOT recorded in APHP from 2015 to 2022.

	2015	2016	2017	2018	2019	2020	2021	2022
Number of proceedings (*n* = 4,858)	562	676	702	675	660	503	548	532
Number of SOT (*n* = 11,324)	1,562	1,552	1,643	1,444	1,486	1,141	1,193	1,303
Number of proceedings in SOT (*n* = 52)	4	5	7	6	7	7	8	8
Proceedings in SOT %	0.71	0.73	0.99	0.88	1.06	1.39	1.45	1.50

### Claims Analysis

Main alleged bases for proceeding after SOT are provided in Table 2.

**TABLE 2:** Main alleged bases for proceedings after SOT (In some cases several complains are alleged).

Organ			Early post-operative complications < 90 days			Late complications > 90 days	Death (covid)
	Iatrogenic complication leading to SOT	Failure to refer in time	Errors in the choice of the graft or in the operative procedure	Alleged failure to diagnose or to treat critical licomplications	Acute neuropathy	Alleged failure to inform and to treat complications	
Heart (*n* = 5)	1	0	1	3	0	1	3
Lung (*n* = 3)	0	1	1	2	0		3 (1)
Liver *(*n* = 19)	3	3	4	10	1	2	13
Kidney **(*n* = 25)							
Recipients (*n* = 19)							
Cadaveric (*n* = 14)	0	0	3	5	7	8	6 (1)
Living (*n* = 5)	1	—	0	2	0	3	3 (2)
Donors: (*n* = 6)	0	—	—	4	4	2	0

*A patient underwent combined liver and kidney transplantation and alleged cruralgia. **One patient underwent a combined pancreas and kidney transplant and died due to a post-operative complication.

Iatrogenic complications leading to SOT were the cause of litigation in five (10.6%) cases including three LT, one CT and one KT. With regard to LT, two cases were the consequence of fulminant hepatitis requiring LT due to the daily postoperative administration of 4 g of paracetamol to malnourished patients. One had good outcome after transplantation, and the other died rapidly of multivisceral failure before being put on the waiting list. The third LT patient had a good outcome after multiple liver abscesses and a biliary fistula due to arterial injury during biliary surgery. Regarding the single iatrogenic complication complaint after CT, a 47-year-old man, developed refractory biventricular dysfunction secondary to aortic aneurysm replacement, underwent emergency transplantation and had a favorable outcome. In the case of KT from a living donor, the recipient, a 35-year-old woman, developed thrombotic end-stage renal failure due to tranexamic acid administration during hemorrhage and a known prothrombic abnormality.

Four SOT candidates died before being put on the waiting list and their family complained of a lost opportunity. The three patients waiting for a liver transplant were a 63-year-old man with sickle cell disease who developed progressive liver and kidney failure leading to death; a 68-year-old man with fulminant hepatitis who died rapidly from multi-organ failure and a 48-year-old man who died of acute hepatitis B infection following a prescription omission. The fourth patient was a 50-year-old woman with pulmonary fibrosis, for whom a transplant was being considered, but who was not listed due to repeated severe episodes of pulmonary sepsis.

Early post-operative complications after SOT were the main causes of litigation. Among the 41 recipients, severe bleeding and septic complications with multi-organ failure were observed in 22 (54%) recipients and led to postoperative death (<90 days) in 12. From the complainant’s perspective, these serious complications were directly attributed to transplant surgery, with a lack of information regarding the use of solid marginal organs in five cases and a technical error during the transplant procedure in four cases. Neuropathy attributed to the surgical procedure was alleged in 12 cases, including plexus nerve in two, one after LT, the other after laparoscopic donor kidney harvesting. Among the 25 patients in the KT group, acute neuropathy with incision pain and femoral sensory and/or motor impairment was alleged in 10 (40%) cases.

Litigation concerning late complications after SOT included: 1) neoplasia with two lymphomas occurring respectively 9 years after LT and 6 years after KT, a Kaposi’s sarcoma 1 year after KT and a fatal fulminant squamous cell carcinoma 2 years after CT; 2) infections with a CMV infection resulting in death recipient 1 year after LT, four deaths attributable to COVID-19, three after KT and one after PT; 3) *de novo* amyloid neuropathy 6 years after LT with domino amyloidosis graft.

The proceedings of the six living kidney donors are presented in Table 3. The sex ratio was 1, the age ranged from 52 to 66 and the donation was made to first-degree relatives in all cases. With the exception of the donor who suffered a brachial plexus stretch during surgery, all complaints were related to the abdominal wall incision, included incisional hernia in two cases and chronic testicular pain in two men. Donors suffering from persistent chronic pain for several years expressed their complaints after the death of the recipient in one case and after financial difficulties in two cases.

**TABLE 3:** Proceedings regarding living kidney donors.

	Sex/age	Date of donation/Procedure	Operative procedure	Proceeding	Recipient/Outcome	Settlement
1	m/64	2008/2021	Laparoscopic	Chronic testicular pain	Spouse/Alive	No
2	f/52	2012/2021	Open	Chronic lower back pain	Child/Dead 2018	No
3	m/52	2015/2016	Laparoscopic	non-medical expanses	Brother/Alive	Yes
4	f/66	2015/2016	Open	Wound dehiscence/incision hernia/non-medical expanses	Son/Alive	Yes
5	f/61	2017/2018	Laparoscopic	Phrenic Para	Sister/Alive	Yes
6	m/54	2019/2022	Laparoscopic	Incisional hernia/chronic testicular pain	Brother/Alive	Yes

Of the 52 cases, 41 (79%) were resolved by verdicts in favour of the defendant without medical malpractice and 11 (21%) in favour of the plaintiff (Table 4).

**TABLE 4:** Main alleged bases for proceeding by verdict type in SOT.

Alleged proceeding	Defendant	Plaintiff	Settlements
Iatrogenic (*n* = 5)	1	4	5
No referral in time (*n* = 4)	2	2	3
Graft choice and technical operative failure (*n* = 9)	6	3	3
ICU management (*n* = 22)	20	2	2
Non-fatal early complication (*n* = 10)	10	0	4
Late complications			
Recurrence (amylose):1	1	0	0
CMV infection *n* = 1	1	0	0
Lymphoma/melanoma *n*= 3	3	0	0
Kaposi (*n* = 1)	1	0	0
Covid (*n* = 4)	4	0	0
Donors (*n* = 6)	6	0	4

Verdicts in favour of the defendant were obtained in 100% of late complications, including COVID-19 deaths, early non-fatal complications and living kidney donors’ procedures. Verdicts were overwhelmingly in favour of the defendant in post-operative management of recipients (90% (*N* = 20) except for two including a suicide of a KT recipient attributed to lack of guardianship and a death by pulmonary embolism attributed to inadequate anticoagulant treatment and in graft selection and operative technical failure [66% (*N* = 6)]. Settlements were awarded for recognised therapeutic risk without medical fault in four donors for non-medical expenses, in four non-fatal early complications, in one iatrogenic transplant that underwent LT due to arterial injury that was considered a surgical therapeutic risk and in one case where the patient was not referred in time. The defendant’s verdicts were associated with settlements paid by ONIAM ranging from 40,000 to 90,000 € for patients who developed non-fatal complications considered to be a therapeutic risk.

Verdicts in favour of the plaintiff were obtained in 11 cases. The categories were as follows all cases of iatrogenic SOT due to medical malpractice with the exception of one case described below, two cases of lack of timely referral due to insufficient information of the patient and his relatives in the case of the sickle cell disease patient who was waiting for a LT and the pulmonary fibrosis patient who was waiting for a PT, three cases blamed the selection of graft or a technical failure, two of which were due to disorganisation of the department, leading to primary non-function attributed to excessive cold ischemia time in one case of KT and to a pulmonary complication attributed to premature discharge; the final case involved a LT performed with a steatosis allograft. All plaintiffs’ verdicts resulted in financial compensation ranging from €110,000 to €1,200,000. The highest amount corresponded to a lifetime pension for a young patient who had undergone LT.

Verdicts were not influenced by the patient’s death: 21/41 (51%) in favour of the defendants compared with 7/11 (63%) in favour of the plaintiffs (*p* = 0.831).

## Discussion

All legal proceedings related to healthcare provided at the AP-HP are grouped together and handled by a specific unit, which has made it possible to collect all proceedings related to SOT. This has made it possible to draw up the first assessment of the nature and development of litigation related to SOT in one of Europe’s largest university hospital centres. This series of 52 cases collected over the last few years showed that transplantation in France is also affected by an increase in litigation, in line with trends observed in the rest of medical society [7–9]. The small number of series published seems somewhat surprising. This is because organ transplantation is a complex operation, involving multiple technical procedures and several medical teams, and is carried out under time-sensitive conditions, which increases the risk of medical malpractice. The increase in the number of SOT-related complaints observed over the study period was not associated with an overall increase in the total number of procedures or an increase in the number of SOTs. Several factors may explain this result.

Firstly, the pandemic of COVID-19 and its high lethality in transplant patients, as shown by our 25% of causes of complaint in the event of death [10]. Intra-hospital contamination was blamed in all cases by the family, but the impossibility of establishing with certainty the contagion and the lack of knowledge about preventive measures resulted in verdicts with no responsibility for the establishment. The second factor is the existence in France of a law offering the possibility of compensation for all victims of a serious medical accident involving a therapeutic hazard [11]. During discussions before the court, we noted that this highly complex activity was not fully understood by families and lawyers. The high expectations of some families to obtain substantial financial compensation led some plaintiffs to question the surgical technique, the medical expertise and the occurrence of well-known long-term complications such as lymphoma [12]. The verdict rate in favour of the plaintiffs was low, around 20%, and logically concerned patients who had undergone SOT after a failure or a deviation from recommended practices and patients for whom a lack of information had been proven. In fact, the occurrence of fulminant hepatitis after intra-hospital administration of paracetamol warned against standardised prescribing in low-weight patients who had been fasting for a long time [13]. In the case of kidney transplantation, the time elapsed between registration on the waiting list and transplantation can be long, more than 5 years, and physicians should re-inform periodically potential kidney recipients about the complications of transplantation and repeat over and over again that transplantation does not mean a cure for the disease, but only a change in the disease.

Throughout the world, organ transplantation remains limited by the insufficient availability of grafts, which makes access to transplantation difficult, and we can expect an increase in complaints about organ allocation [14]. In France, around 5,000 deaths of patients on the waiting list were reported during the period covered by this study. The surprising absence of litigation concerning this category of patients can be seen as an adherence to the rules laid down in our country by the ABM. These rules, drawn up by our state agency, are established and regularly revised in collaboration with the transplant community, and explained to future recipients and their relatives by the medical team and the coordinating nurses [15]. During the legal debates in this series, the quality of the information provided by this group of advanced practice nurses was never called into question. On the other hand, the inadequate quality of the information provided by the medical team to the patient and his relatives has often been criticised and judicially sanctioned, as illustrated by the plaintiff’s verdict in the case of a medical contraindication to inclusion on the waiting list, which had not been sufficiently communicated to the family. However, no conclusions could be drawn, as patients and their families may have different expectations of the medical team and the coordinating nurses.

Indeed, the inadequacy of information shared and recorded in the presence of the patient and their relatives throughout the organ transplantation process is a key factor in the analysis of this series [16]. The high rate and fatal risk of post-transplant complications highlights the need to share information and knowledge at a time when recipients are becoming older and have more co-morbidities, increasing the possibility of receiving high-risk organs [17]. In this context, the large number of people with different levels of expertise involved can make it difficult to understand patient care and the risks involved. Our results suggest that patients and their families should be given more information at all stages of SOT, and that this information and major decisions should be traceable throughout the transplantation process in the transplant units.

One of the main causes of serious post-transplant complications is organ failure immediately after transplantation, associated with the use of so-called extended criteria grafts. This study revealed that none of the patients or their families were aware of the risk associated with these transplants. This lack of information may be justified from a legal and ethical point of view [18]. In fact, it has been shown that most patients undergoing long-term transplantation wanted to be informed and involved in the decision at the time of organ proposal regarding the risks associated with the donor [19]. A marginal transplant is always accepted by clinicians with a reasonable degree of safety, but it may be judicially deemed to be defective, i.e., it does not offer the safety that a person is entitled to expect [20]. Although only two verdicts in this series have called into question the information relating to the transplant, it is probably reasonable to introduce specific consent in France concerning the risks associated with the donor, along the lines of what is practised in the United Kingdom [16].

The majority of cases in this series illustrate the high level of KT activity in France, with around 3,500 cases per year. While living donor KT (LDCT) accounts for 15% of KT in France, more than 40% of the KT cases included in this series involved a procedure involving a living donor. Although LDKT is associated with better outcomes for the recipient than deceased organ donation, the high rate of legal disputes reported here illustrates a singular aspect of living organ donation [21]. Indeed, the complications and failure of living organ donation are often associated with the donor’s guilt over the failure of this gift. Even in the event of a favourable outcome for the recipient, disputes with donors could reflect the profound and complex impact of organ donation by living people [22]. Having been a saviour, they have to get used to their vulnerability due to the absence of the donated organ [23]. The relationship with the beneficiary, their social environment and the medical system is strongly affected by frequent and constant disappointment in relation to what they expected from their donation. It would be worth highlighting the need for better attention and follow-up for donors, many of whom feel neglected too quickly. One of the original features of this study is that it brought together the legal proceedings brought by six donors against the institution. In both the laparoscopic and open approaches, the alleged complications were attributed to abdominal wall complications, including chronic testicular pain, which is often overlooked in men [24, 25]. In this series, complications related to donations are often associated with non-medical expenses, which explains why some settlements have been awarded despite the absence of fault or negligence. The principle of financial neutrality applies to donations, which means that they are free of charge. The results of this series confirm that these complications and their potential impact are not detailed and that a standardised informed consent form specific to nephrectomy from a living donor is strongly recommended [16]. We could also suggest improving the psychological assessment of the living donor before and after the operation in order to limit donor disappointment after the transplant and the feeling of being abandoned.

The main limitation of this study is the exclusive selection of proceedings aimed at obtaining financial compensation. Several claims that were resolved by local mediation without settlement were not included. Although the number of cases presented is significant, it cannot be ruled out that some proceedings are resolved quickly and confidentially, perhaps to minimise media coverage in order to protect the public image of occupational therapy and/or the reputation of the hospital/staff involved.

## Conclusion

This study has shown that transplantation activity in France is also affected by the trend towards increased litigation against the medical community. Although no liability was found against the institution in almost 80% of the verdicts, certain major trends should be taken into account in order to maintain this activity and slow down or reduce the rate of litigation. One of the main recommendations is to improve the quality of information provided to patients and their relatives about the risks of emergency surgery, and the development and treatment of complications. The second is to provide, where appropriate, information on the specific risk to the donor, which should be in line with what is done in many other countries. Improving the information and psychological assessment of living donors is essential if the technique of transplantation is to be sustainable, given its excellent overall results.

## Data Availability

The raw data supporting the conclusion of this article will be made available by the authors, without undue reservation.

## Footnotes

1
www.agence-biomedecine.fr
